# Autoinflammatory diseases: a Latin American multicenter study according to age and sex

**DOI:** 10.1590/1984-0462/2024/42/2022184

**Published:** 2023-10-23

**Authors:** Daniela Gerent Petry Piotto, Katia Kozu, Nádia Emi Aikawa, Pedro Lopes Carneiro, María Martha Katsicas, Sheila Knupp Feitosa de Oliveira, Taciana de Albuquerque Pedrosa Fernandes, Claudia Saad Magalhães, Ana Luiza Garcia Cunha, Blanca Elena Rios Gomes Bica, Carlos Nobre Rabelo, Cristina Battagliotti, Erica Naomi Naka Matos, Flavia Patrícia Sena Teixeira Santos, Flavio Roberto Sztajnbok, Liliana Bezrodnik, Marcia Bandeira, Marta Cristine Felix Rodrigues, Pablo García Munittis, Simone Appenzeller, Teresa Cristina Martins Robazzi, Gleice Clemente, Clovis Artur Silva, Maria Teresa Terreri

**Affiliations:** a Universidade Federal de São Paulo, SP, Brazil.; b Instituto da Criança e do Adolescente, Hospital das Clínicas, Faculdade de Medicina, Universidade de São Paulo, SP, Brazil.; c Universidade Federal de São Paulo, São Paulo, SP, Brazil.; d Hospital Juan P. Garrahan, Buenos Aires, Argentina.; e Universidade Federal do Rio de Janeiro, Rio de Janeiro, RJ, Brazil.; f Hospital das Clínicas de Botucatu, Universidade Estadual Paulista, Botucatu, SP, Brazil.; g Hospital Infantil João Paulo II, Belo Horizonte, MG, Brazil.; h Hospital Geral de Fortaleza, Fortaleza, CE, Brazil; i Hospital de Niños Dr. Orlando Alassia, Santa Fé, Argentina.; j Universidade Federal de Mato Grosso do Sul, Campo Grande, MS, Brazil.; k Universidade Federal de Minas Gerais, Belo Horizonte, MG, Brazil.; l Centro de Inmunología Clínica, Buenos Aires, Argentina.; m Hospital Pequeno Príncipe, Curitiba, PR, Brazil.; n Hospital El Cruce, Buenos Aires, Argentina.; o Universidade de Campinas, Campinas, SP, Brazil.; p Universidade Federal da Bahia, Salvador, BA, Brazil.

**Keywords:** Autoinflammatory disease, Children, Adolescents, Familial Mediterranean fever, Periodic fever, Doença autoinflamatória, Crianças, Adolescentes, Febre familiar do Mediterrâneo, Febre periódica

## Abstract

**Objective:**

To evaluate autoinflammatory diseases (AID) according to age at diagnosis and sex, and response to therapy in a large population.

**Methods:**

This is a cross-sectional observational study of a Latin American registry using a designed web system for data storage, collected between 2015 and 2018. Any altered findings during follow-up were recorded. The forms were translated into Portuguese and Spanish, including demographic, clinical, laboratory, genetic and treatment characteristics.

**Results:**

We included 152 patients, 51.3% male and 75% Caucasian. The median age at disease onset was 2.1 years (0–15.6 years) and median age at diagnosis 6.9 years (0–21.9 years); 111 (73%) were children (0–9 years old), and 41 (27%) were adolescents and young adults (AYA) (10–21 years old). Periodic fever, aphthous stomatitis, pharyngitis, and adenitis syndrome (PFAPA) occurred in 46/152 (30%), chronic non-bacterial osteomyelitis (CNO) in 32/152 (21%), and familial Mediterranean fever (FMF) in 24/152 (15.7%). PFAPA was significantly higher in young children than in AYA (38.7% vs. 7.3%, p<0.001), while CNO were lower (13.5% vs. 41.5%, p<0.001). The frequency of females was significantly higher in CNO (28.4% vs. 14.1%, p=0.031) and lower in FMF (8.1% vs. 23.1%, p=0.011). The most used drugs were glucocorticoids, non-steroidal anti-inflammatory drugs (NSAID), and colchicine. Glucocorticoids and colchicine treatment were used in all AID with good to moderate response. However, cryopyrin-associated periodic syndromes (CAPS) seemed unresponsive to glucocorticoids. NSAIDs and methotrexate were the main medications used to treat CNO.

**Conclusions:**

Differences among AID patients were observed in the LA population regarding sex and age at disease diagnosis.

## RESUMO

**Objetivo:**

Avaliar as doenças autoinflamatórias (DAI) de acordo com sexo e idade no momento do diagnóstico e a resposta terapêutica em uma grande população.

**Métodos:**

Este é um estudo observacional transversal de um registro latino-americano que usou um sistema de dados coletados entre 2015 e 2018. Quaisquer achados alterados ao longo do acompanhamento foram registrados. Os formulários foram traduzidos para os idiomas português e espanhol, incluindo características demográficas, clínicas, laboratoriais, genéticas e de tratamento.

**Resultados:**

Incluímos 152 pacientes, sendo 51,3% do sexo masculino e 75% da raça branca. A média de idade de início da doença foi de 2,1 anos (0–15,6 anos) e a média de idade de diagnóstico 6,9 anos (0–21,9 anos); 111 (73%) eram crianças (0–9 anos) e 41 (27%) adolescentes/adultos jovens (10–21 anos). A síndrome de febre periódica, estomatite aftosa, faringite e adenite (PFAPA) ocorreu em 46/152 (30%), osteomielite não bacteriana crônica (CNO) em 32/152 (21%) e febre familiar do Mediterrâneo (FMF) em 24/152 (15,7%). A PFAPA foi significativamente maior em crianças pequenas (38,7 *vs*. 7,3%, p<0,001), e a CNO, em adolescentes/adultos jovens (13,5 *vs.* 41,5%, p<0,001). A frequência do sexo feminino foi significativamente maior na CNO (28,4 *vs*. 14,1%, p=0,031) e menor na FMF (8,1 *vs*. 23,1%, p=0,011). Os medicamentos mais utilizados foram glicocorticoides, anti-inflamatórios não esteroidais (AINE) e colchicina. O tratamento com glicocorticoides e colchicina foi usado em todas as DAI com resposta boa a moderada. No entanto, as síndromes periódicas associadas à criopirina (CAPS) pareciam não responder aos glicocorticoides. AINE e metotrexato foram os principais medicamentos utilizados no tratamento da CNO.

**Conclusões:**

Diferenças de pacientes com DAI foram observadas na população latino-americana em pacientes agrupados por sexo e idade ao diagnóstico da doença.

## INTRODUCTION

Autoinflammatory diseases (AID), or recently renamed as human inborn errors of immunity, are rare diseases with clinical manifestations characterized by recurrent episodes of systemic inflammation that have been described mostly over the last 20 years. Although there has been a longstanding awareness of familial Mediterranean fever (FMF) in the Middle East, its monogenic transmission was first described in 1997. FMF is the most prevalent monogenic AID globally.^1–4^

In 2022, a comprehensive revision of the new definition was published, and two major categories were defined, type 1 interferonopathies and defects affecting the inflammasome; additionally, a third group was classified as non-inflammasome related conditions.^1^

Multicenter studies are relevant in characterizing clinical and genetic profiles of these diseases among different populations and ethnic backgrounds. Indeed, many efforts have been made for the better understanding of prevalence and clinical features in Europe (Eurofever), and in the United States and Canada (Childhood Arthritis and Rheumatology Research Alliance — CARRA).^4,5^

All previous Latin American (LA) studies of AID were limited to case reports or small case series in three countries: Brazil,^6–12^ Argentina and Chile.^13–16^ In 2012, Jesus et al. described the Brazilian prevalence and characteristics of 102 patients, who were systematically evaluated for AID, and they found cryopyrin-associated periodic syndromes (CAPS), tumor necrosis factor receptor-associated periodic syndrome (TRAPS), and FMF as the most frequent.^17^ In this study, approximately one third of the Brazilian patients with a clinical suspicion of AID had a confirmed genetic test, and 7 of the 26 different identified mutations were novel.^17^ However, these were mainly descriptive studies and differences between sex and age groups have not been explored yet and there is still no robust data of real-world practice among the LA population.

Therefore, the objective of the present study was to contribute to alert pediatricians to AID diagnosis due to the high morbidity in childhood. We also aimed to evaluate frequencies of AID according to age at diagnosis and sex, and response to therapy in a large population.

## METHOD

This is an observational, descriptive, multicenter study of a LA registry based in a web system for data collection of AID clinical profiles. We invited pediatric rheumatologists from 11 LA countries to participate in this study. Herein we reported the results of the first 152 patients enrolled in two participating countries (Brazil and Argentina) from 2015 to 2018.

The inclusion criteria were: children and adolescents and young adults (AYA) up to 21 years old with AID with at least six months of follow-up, with first symptoms at onset suggestive of AID (periodic fever, multi-organ involvement, polyserositis). Symptoms should be diagnosed by either typical clinical features and genetic positive testing; or, typical clinical features without positive genetic testing (due to lack of known genetic mutations or due to failure in finding the genetic confirmation); or, with mild and/or atypical clinical features or even absence of symptoms and familiar background of AID and genetic confirmation.^4^ The patients were classified according to the current classification of AID.^1^ In the defects affecting the inflammasome group FMF,^4,10^ mevalonate kinase deficiency/hyperimmunoglobulin D syndrome (MKD/HIDS),^4,10^ and CAPS^4,10^ were included. In the type 1 interferonopathies, stimulator of interferon genes (STING)-associated vasculopathy with onset in infancy (SAVI)^18^ and deficiency of adenosine deaminase 2 (DADA2)^19^ were included. A third group classified as non-inflammasome related conditions included TRAPS,^4,10^ periodic fever, aphthous stomatitis, pharyngitis, and cervical adenitis syndrome (PFAPA)^4,5,10^ pediatric granulomatous arthritis (PGA),^4,9^ chronic non-bacterial osteomyelitis (CNO),^4,20^ pyogenic arthritis, pyoderma gangrenosum and acne syndrome (PAPA),^4,21,22^ pyogenic arthritis, acne, pyoderma gangrenosum and suppurative hidradenitis (PAPASH),^21,22^ deficiency of IL-36 receptor antagonist (DITRA),^4,21^ deficiency of IL-1 receptor antagonist (DIRA),^4,21^ chronic atypical neutrophilic dermatosis with lipodystrophy and elevated temperature syndrome (CANDLE).^23^ Patients with other causes of symptoms, such as chronic autoimmune diseases, infection (proved by culture or polymerase chain reaction to infectious agents or eventually presumptive diagnosis), and neoplasia were excluded.

The attending physicians provided demographic data, age at diagnosis, and all the clinical and laboratorial data up to the time of evaluation. All investigators used the same protocol for data collection using the web system. Laboratorial findings included complete blood count, acute phase reactants, ferritin, albumin, and protein electrophoresis. The abnormal values were considered according to the local laboratory reference values. Each of the centers performed genetic evaluation in a certified laboratory, which was indicated by the attending physician according to the clinical suspicion. We valued only pathogenic and likely pathogenic genetic variants according to the Infevers database (Internet fevers, https://infevers.umai-montpellier.fr/web/).

The treatment was also registered. Disease activity was evaluated according to the opinion of the attending physician. We defined arbitrarily the treatment response as complete response (with resolution of the signs/symptoms and normalization of laboratorial findings within three to six months after the medication introduction), partial response (partial resolution within three to six months), or unresponsive (no response at all).^24^ Remission was defined as absence of signs and symptoms for at least one year. The definition of clinical response was based on the proposed definition for response in PFAPA syndrome, since it is important in clinical practice as a guidance for the specialists in escalating or changing therapy.^5^

The data registry was translated into the Portuguese and Spanish languages and the variables were ethnicity, sex, age at disease onset, age at diagnosis, AID family history, clinical features, laboratorial and subsidiary findings (such as imaging and histopathology), treatment, response to medications, and family genetic profile (in the case of presence of family history). After local Ethical Committee approval, each investigator entered the data into the web system database (Enfermedades Autoinflamatorias — EA Latam.com). The original data source was the medical records from each of the centers. To assure anonymity and confidentiality, codes were assigned to each patient, each center and country.

Statistical analysis: continuous variables were described by median, minimum and maximum values. Comparisons of categorical variables (confirmed diagnosis, signs/symptoms, laboratorial and treatment data) between children and AYA groups and between both sexes were performed using Fisher's exact test or Pearson's chi-square test, and the results presented as number and frequency. Significance levels, for all analyses, were established at 5% and p-value (p)<0.05.

## RESULTS

**Figure 1:**
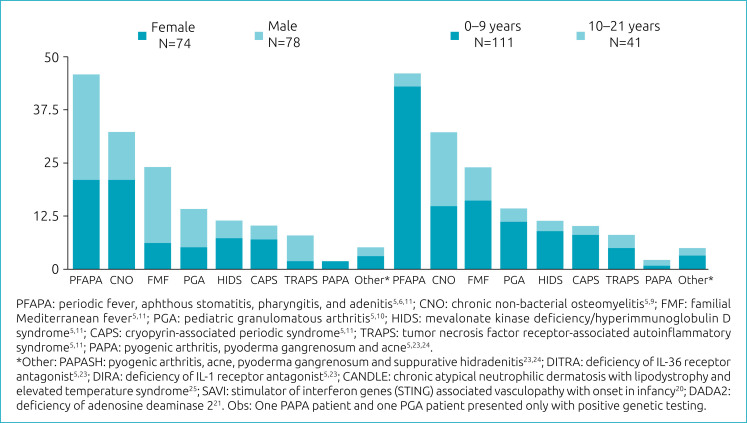
Diagnoses of Latin American patients with autoinflammatory diseases according to age at diagnosis and sex (n=152).

We included 152 patients, of which 51.3% were male, 75% Caucasian, 20% mixed race, 3% Latin American, and 1% Asian (ethnicity was not reported in 1%). The median age at disease onset was 2.1 years (0–15.6 years) and the median age at diagnosis was 6.9 years (0–21.9 years); 111 (73%) were diagnosed between 0 and 9 years old and 41 (27%) between 10 and 21 years old. The most frequent AIDs were: PFAPA, CNO and FMF. PFAPA was significantly higher in young children than AYA (38.7% vs. 7.3%, p<0.001), while CNO were lower (13.5% vs. 41.5%, p<0.001). The frequency of females was significantly higher in CNO (28.4% vs. 14.1%, p=0.031) and lower in FMF (8.1% vs. 23.1%, p=0.011) (Figure 1).

The diagnosis was based only on clinical confirmation in 98 (64.5%) patients, 78 of them presented PFAPA or CNO according to the respective criteria, and those remaining were considered as suggestive of AID. The diagnosis was based on clinical and genetic confirmation in 52 (34.2%) patients and based only on genetic test confirmation in 2 (1.3%).

The main signs and symptoms of AID were fever, malaise, arthralgia, limb and abdominal pain, cervical lymphadenitis, and aphthous stomatitis. Some clinical features were more frequent in children, such as fever, cervical lymphadenitis, aphthous stomatitis, pharyngitis, and generalized lymphadenopathy. Others were more prevalent in AYA patients, such as arthralgia, arthritis, bone pain, osteomyelitis, osteitis, and aseptic arthritis. Bone pain, osteomyelitis, and osteitis were more frequent in girls, while weight loss was more common in boys. The most frequent laboratory findings were high erythrocyte sedimentation rate (ESR) and/or C-reactive protein (CRP), leukocytosis, thrombocytosis, and anemia. Most of them do not differ when compared by age at diagnosis and sex, except for leukocytosis and thrombocytosis, which occurred more frequently in children, and elevated ferritin, more frequent in boys.

**Figure 2:**
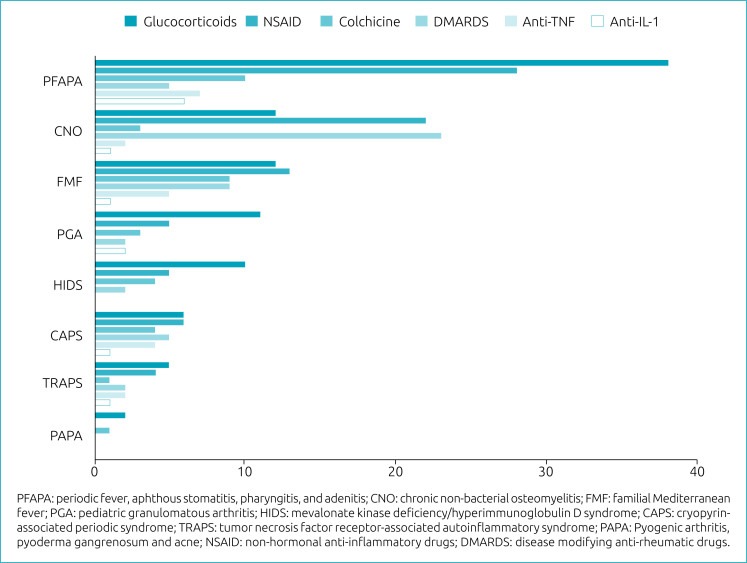
Frequency of drug treatment in Latin American patients with autoinflammatory diseases (n=152).

The most used drugs were glucocorticoids, non-steroidal anti-inflammatory drugs (NSAID) and colchicine. Glucocorticoids and colchicine treatment were used in all AID with good to moderate response. However, CAPS seemed unresponsive to glucocorticoids. NSAIDs and methotrexate were the main medications used to treat CNO (Figure 2).

Nine (19.6%) PFAPA patients with moderate or no response to systemic therapy were submitted to tonsillectomy intervention, with complete response in seven of them.

Remission was observed in 76 (50%) patients (50% of PFAPA, 65.6% of CNO, 75% of FMF, 21.4% of PGA, 18.2% of HIDS, 40% of CAPS, 37.5% of TRAPS, and 50% of PAPA). Amyloidosis was not observed in any patient. Macrophage activation syndrome (MAS) occurred in two patients, one with HIDS, and one with PGA. Mortality rate was 1.3%. Of the two deaths, one patient had CAPS (Chronic Infantile Neurological Cutaneous Articular/ Neonatal Onset Multisystem Inflammatory Disease — CINCA/NOMID) and the other had PGA complicated by MAS.

## DISCUSSION

This is the first assessment report of AID patients from the LA registry. The comparison of the clinical presentation according to age at diagnosis, and sex was evaluated. In the present study, we observed PFAPA as the most frequent AID, followed by CNO and FMF.

We noticed a diagnosis delay of about five years, reflecting the lack of knowledge and inability to recognize these diseases, especially by general pediatricians who are the first to follow these patients with AID.

PFAPA was observed more frequently in children, between 0 and 9 years of age, which has been described in the literature; however, it is known that PFAPA tends to improve over time, and bias related to this cannot be excluded.^24^ In addition, it is also known that PFAPA is very rare before one year of age. On the other hand, CNO occurred more frequently in AYA patients, between 10 and 21 years old. We observed a prevalence of females in CNO patients, corroborating Hedrich et al. study, who also described a slight female predominance.^25^ As stated in previous reports, a higher prevalence of CNO was described in young age patients.^20^ FMF predominated in boys as observed by other authors.^16,17^

Some clinical AID features were more common in children and others were more frequent in AYA patients. These were probably due to the prevalence of PFAPA in children and the prevalence of CNO in AYA.^24,25^ Differences in sex were not observed in our study except for weight loss (more common in boys) and bone pain, osteomyelitis, and osteitis (more common in girls).

Leukocytosis and thrombocytosis that occurred more frequently in children, and elevated ferritin, that was more frequent in boys, were the only laboratory findings that showed differences between the groups. We could not find in the literature any difference of sex and age prevalence related to cumulative signs/symptoms and laboratorial findings.

Variants of undetermined significance (VUS) are very common in clinical practice and often lead to erroneous conclusions when analyzed separately. In order not to value misdiagnosis we analyzed carefully the Infevers database. We followed many patients without confirmatory genetic testing due to the absence of a known mutation (as in PFAPA and CNO), or due to the unavailability of testing. Although genetic evaluation is an extremely important tool for the diagnosis of AID, in real-life practice in our centers, the genetic testing is still a challenge for its costs and lack of health insurance coverage within the national health systems of both countries. The study of Gattorno et al., suggested new classification criteria for AID, with or without genetic testing, in cases of typical clinical features.^4^ We consider this new classification important to support our practice in the LA population.

Although most AID have a genetic mutation already described, PFAPA mutation is still being investigated. Despite the higher frequency of PFAPA compared to other AID, some differential diagnoses must be ruled out, especially in the presence of skin lesions, severe gastrointestinal symptoms, and symptoms in patients younger than one year old — all of them very uncommon in PFAPA.

Recent advances in the understanding of the molecular and pathophysiological basis of AID led to new treatment strategies. Brazilian evidence-based guidelines for treatment were published in 2016.^26–28^ Patients with periodic fever syndromes clearly benefit from anti-interleukin (IL) 1 treatment.^26^ Colchicine remains the mainstay for FMF treatment and represents the second line therapy for PFAPA.^27,28^ Adequate therapeutic resources are scarce for the rare monogenic conditions, which can be subject to rational therapy with tumor necrosis factor (TNF) and IL-1 antagonists. In LA countries, the availability and prescription of anti-IL1 is indeed very limited. Because of this, glucocorticoids may be the only therapeutic option for many patients.

Glucocorticoids and colchicine treatment were used in all described diseases in our series with good to moderate response. However, CAPS seemed unresponsive to glucocorticoids. The management of PFAPA syndrome is very heterogeneous since evidence-based treatment guidelines are lacking. NSAIDs and methotrexate were the main medications used to treat CNO, as stated in the published guidelines.^29^

Our study strengths rely on real-life reports from 20 centers in two countries, comparing two age groups (children and AYA) at diagnosis and follow-up in the LA population. We acknowledge the limitations due to genetic tests’ unavailability for their costs, lack of health insurance coverage, no identification of *de novo* mutations in our patients, and the unavailability of anti-IL1 treatment for most participating centers. Likewise, the retrospective data collection, the lack of use of validated disease monitoring tools, such as autoinflammatory diseases activity index (AIDAI), and the lack of more accurate exams for monitoring amyloidosis, were among the limitations.^30^ Additionally, PFAPA tends to improve over time, and bias related to this cannot be excluded. The LA AID group took its first steps by including patients from two neighboring countries, but extending data collection to all countries in such a large region and population is a challenge.

Pediatricians must be aware of these new diseases with various clinical manifestations, such as fever of unknown origin associated with inflammatory parameters, recurrent episodes of aphthous lesions, pharyngitis, and lymphadenitis (like in PFAPA), or recurrent bone pain (like in CNO). The correct referral to a specialist can lead to the appropriate treatment, improving the prognosis.

Future studies by our group targeting the LA population, have been planned to expand the registry, besides prospectively evaluating physical growth, puberty milestones, and health-related quality of life. It is expected that through this network of professionals and collaborating centers, we can further raise awareness among pediatricians and pediatric rheumatologists for the early recognition of these diseases in different countries.

## Data Availability

The database that originated the article is available in an open repository (web database EA LATAM.com), or upon request with the corresponding author.
